# Exposure profile of mercury, lead, cadmium, arsenic, antimony, copper, selenium and zinc in maternal blood, cord blood and placenta: the Tohoku Study of Child Development in Japan

**DOI:** 10.1186/s12199-019-0783-y

**Published:** 2019-05-17

**Authors:** Miyuki Iwai-Shimada, Satomi Kameo, Kunihiko Nakai, Kozue Yaginuma-Sakurai, Nozomi Tatsuta, Naoyuki Kurokawa, Shoji F. Nakayama, Hiroshi Satoh

**Affiliations:** 1 Centre for Health and Environmental Risk Research, National Institute for Environmental Studies, Tsukuba, Ibaraki Japan; 2 Department of Nutrition, College of Nutrition, Koshien University, Takarazuka, Hyogo Japan; 3 Department of Development and Environmental Medicine, Tohoku University Graduate School of Medicine, Sendai, Miyagi Japan; 4 Department of Human Health and Nutrition, Faculty of Comprehensive Human Sciences, Shokei Gakuin University, Sendai, Miyagi Japan; 5 Miyagi University of Education, Sendai, Miyagi Japan; 6 Tohoku University, Sendai, Miyagi Japan

**Keywords:** Cord blood, Placenta, Maternal blood, Trace element, Metal

## Abstract

**Background:**

The effects of prenatal exposure to toxic elements on birth outcomes and child development have been an area of concern. This study aimed to assess the profile of prenatal exposure to toxic elements, arsenic (As), bismuth (Bi), cadmium (Cd), mercury (total mercury (THg), methylmercury (MHg), inorganic mercury (IHg)), lead (Pb), antimony (Sb) and tin (Sn), and essential trace elements, copper (Cu), selenium (Se) and zinc (Zn), using the maternal blood, cord blood and placenta in the Tohoku Study of Child Development of Japan (*N* = 594–650).

**Methods:**

Inductively coupled plasma mass spectrometry was used to determine the concentrations of these elements (except mercury). Levels of THg and MeHg were measured using cold vapour atomic absorption spectrophotometry and a gas chromatograph-electron capture detector, respectively.

**Results:**

Median concentrations (25th–75th) of As, Cd, Pb, Sb, Sn and THg in the maternal blood were 4.06 (2.68–6.81), 1.18 (0.74–1.79), 10.8 (8.65–13.5), 0.2 (0.06–0.40) and 0.2 (0.1–0.38) ng mL^−1^ and 5.42 (3.89–7.59) ng g^−1^, respectively. Median concentrations (25th–75th) of As, Cd, Pb, Sb, Sn and THg in the cord blood were 3.68 (2.58–5.25), 0.53 (0.10–1.25), 9.89 (8.02–12.5), 0.39 (0.06–0.92) and 0.2 (0.2–0.38) ng mL^−1^ and 9.96 (7.05–13.8) ng g^−1^, respectively.

**Conclusions:**

THg and Sb levels in the cord blood were twofold higher than those in the maternal blood. Cord blood to maternal blood ratios for As, Cd and Sb widely varied between individuals. To understand the effects of prenatal exposure, further research regarding the variations of placental transfer of elements is necessary.

**Electronic supplementary material:**

The online version of this article (10.1186/s12199-019-0783-y) contains supplementary material, which is available to authorized users.

## Background

Humans have experienced various adverse effects from environmental contaminants, especially toxic elements such as arsenic (As), cadmium (Cd), mercury (Hg) and lead (Pb), which are detrimental to human health. In addition, these elements are ranked among the ten most toxic substances by the Agency for Toxic Substances and Disease Registry [1]. Although individuals in developed countries may no longer suffer from severe poisoning caused by exposure or overexposure to these elements, chronic and low exposure remains a health hazard. A developing foetus, in particular, is highly vulnerable to the toxic effects of these elements [2, 3].

Maternal exposure to these toxic elements may happen through diet, air, drinking water, house dust and tobacco use and/or passive smoking [3]. Mothers may also be exposed to these elements in the workplace. Inorganic Hg (IHg) is biologically transformed in the aquatic environment from its inorganic form into methylmercury (MeHg), a toxic form of the element. Consequently, humans who consume fish and other seafood are potentially exposed to MeHg, and this exposure increases the risk of neurodevelopmental disorders [2, 4, 5]. Exposure to Pb can cause spontaneous abortions [6] and can lead to reduced birth weight, gestational hypertension, congenital malformations [7] and impaired neurodevelopment [8]. Even low levels of in utero exposure to Pb may result in adverse birth outcomes and neurodevelopmental effects such as reduced intelligence and symptoms pertaining to attention-deficit/hyperactivity disorder (ADHD) [9, 10]. Exposure to Cd is also potentially hazardous to foetal health. However, the placenta acts as a barrier to Cd exposure for the foetus by increasing metallothionein expression [11]. Nonetheless, Cd is found in the cord blood, and this exposure has been associated with decreased birth weight [12]. Prenatal As exposure is associated with adverse birth outcomes [13], and it can adversely affect the health of adults [14] and can lead to increased mortality and an increased risk of lung and liver cancers [15, 16]. Copper (Cu), zinc (Zn) and selenium (Se) are essential trace elements and involved in numerous biochemical processes that support life [17]. Bismuth (Bi), a minor metal, has been used in pharmaceuticals and cosmetics. Bi is also found in low concentrations in biological and environmental samples, including blood, urine, food and water [18, 19]. Antimony (Sb) is another minor metal and used in pharmaceuticals. Sb is usually present in the environment in very low concentrations [20]. Volonakis et al. [21] reported the development of new Pb-free materials (e.g. Pb-free inorganic halide double perovskites) based on Bi or Sb. New materials made with Bi and Sb have been used as a substitute for lead. According to a review of the literature, the available data regarding the effects of exposure to Sb and Bi on general and vulnerable populations is insufficient.

The effects of prenatal exposure to toxic elements have been reported extensively. Consequently, more attention has been paid to the effects of toxic elements on pregnancy outcomes and/or adverse developmental effects at levels lower than current international guidelines [22–24]. The extent of prenatal exposure to environmental contaminants has been assessed primarily using cord and/or maternal blood samples [25–27]. However, few reports have assessed the relationship between ten elements by using the complete data of the maternal blood, cord blood and placenta in general populations of Japan.

In this study, we presented a novel examination of maternal exposure to multiple elements [i.e. Cd, Hg (total Hg [THg], MeHg and IHg), Pb, As (total As), Sb, Bi, Sn, Cu, Zn and Se] and transplacental transfer of these elements from the mother to the foetus in Japan. We investigated these elements by considering the environmental health effects, the possibility of minor metal exposures and the confounding aspect of trace elements. The aim of this study was to (1) evaluate the prenatal exposure to toxic and essential trace elements using the maternal blood and cord blood by focusing on As, Bi, Cu, Cd, Hg (THg, MeHg and IHg), Pb, Sb, Se, Sn and Zn and (2) assess the placental transfer of the selected elements.

## Methods

### Study design, subjects and sampling

We performed a birth cohort study called the Tohoku Study of Child Development (TSCD) in the northeastern area of Japan. The Japanese refer to this area as the ‘Tohoku region’. This study was conducted in an urban area (registered between 2001 and 2003) and a coastal area (registered between 2002 and 2006). The details and protocols of this study have been described elsewhere [28]. In summary, 687 pregnant Japanese women were enrolled for participation in this study, and we obtained written informed consent from all participants before beginning their part in the study [28]. We followed up on the mothers and their resulting infant pairs, and the infants’ development was examined regarding prenatal exposures to environmental contaminants, such as MeHg, Pb and polychlorinated biphenyls (PCBs) [29–32]. This article analysed the samples from the urban area (Additional file 1: Figure S1).

In the urban area, the maternal blood was collected at 28 weeks of pregnancy using venepuncture into a tube containing heparin. Similarly, the cord blood was collected from the umbilical cord vein immediately after delivery; the placentas were also collected during delivery. A representative sample was collected from the lower parts of the root of the cord tissue because the placenta is a large organ and a heterogeneous mixture of placental cells and decidual tissues containing the maternal and foetal blood [33]. Acharya et al. indicated that approximately 40% of the blood is contained in the placenta. The representative sample was processed by homogenisation before use. The blood and placenta samples were stored at − 80 °C until the analyses.

### Analytical methods

#### Determination of mercury

Cold vapour atomic absorption spectrometry (CVAAS; HG-201, Sanso Seisakusho Co. Ltd., Tokyo, Japan) was used to measure the THg level in the whole blood and placenta. The analytical CVAAS method has been completely described elsewhere [34, 35]. A gas chromatograph-electron capture detector was used to measure the MeHg level in the blood samples (GC-ECD) [34, 36]. The concentrations of IHg were calculated by subtracting MeHg levels from THg concentrations.

#### Determination of other toxic metals and essential trace elements

We determined the levels of toxic elements As, Bi, Cd, Pb, Sb and Sn and the levels of essential trace elements Cu, Zn and Se using inductively coupled plasma mass spectrometry (ICP-MS; 7500c, Agilent Technologies, Inc. CA, USA, Table 1). A sample (approximately 0.5 mL of the blood and approximately 0.2 g of the placenta) was weighed and placed at the bottom of a fluororesin airtight sample container. Appropriately 1 mL of nitric acid (HNO_3_) was added to the blood, whereas 2 mL of HNO_3_ and 2 mL of distilled water were added to the placenta. Pressure decomposition was conducted using a microwave at 600 W for 30 min (MDS-2000, CEM Corporation, NC, USA). These analyses were performed by IDEA Consultants, Inc. (Tokyo, Japan).

**Table 1:** Instrumental setting for ICP-MS

Instrument	Agilent 7500c
Mass monitored	Cu: 63
	Zn: 66
	As: 75
	Se: 78
	Cd: 111
	Sn: 118
	Sb: 121
	Pb: 208
	Bi: 209
Internal standards	Ge: 72
	Y: 89
	Rh: 103
	In: 115
	Tl: 205
Reaction gas	He, H_2_
RF power	1.5–1.6 kW

### Analytical quality control

We used a reference material (Seronorm Trace Elements Whole Blood Level 2 and 3 prepared by the SERO AS, Norway) for quality control (Additional file 1: Tables S1 and S2). Moreover, the data quality for THg, Cd and Pb concentrations was verified using external quality assurance programmes (Additional file 1: Table S2). The quality of MeHg analyses was confirmed in two laboratories by IDEA Consultants, Inc. and International Mercury Laboratory, Co., Ltd. Both laboratories used the same whole blood samples (*N* = 5, Pearson’s *r* = 0.999, *P* < 0.001, Additional file 1: Figure S2). The limits of detection (LODs) for each analyte were calculated according to Currie’s method [37].

### Data analysis

The concentration levels of the elements in the maternal blood, cord blood and placenta were assessed for normality using the Shapiro-Wilk test. Their concentrations were presented as medians, namely, 25–75 percentiles and ranges, because the distribution was skewed. We also performed Spearman’s rank correlation coefficients (*rho*) analysis. The concentration levels of the samples were assessed by performing a nonparametric Kruskal-Wallis analysis; this was followed by a Dunn test. *P* < 0.05 was considered statistically significant. The software package JMP12.0.2 (SAS Institute Inc., Cary, NC, USA) was used for statistical analysis.

## Results

The toxic and trace element concentrations are summarised in Table 2 (*N* = 594–650). The mean gestational age (SD) and maternal age (SD) at birth were 39.5 (1.3) weeks and 31.4 (4.4) years old, respectively (*N* = 580, Additional file 1: Figure S1). The mean placental weight (SD) at birth was 559 (97) g (*N* = 565). Sn and Bi levels were 44% and 7%, respectively, in the maternal blood sample and 36% and 18%, respectively, in the cord blood sample. Therefore, Bi and Sn concentration levels were excluded from the subsequent analysis.

**Table 2:** Exposure levels of toxic and essential trace elements in the maternal blood, cord blood and placenta

	Elements	*N*	Median	25th–75th percentile	Min	Max	< LOD^b^ (%)
Maternal blood (ng mL^−1^)	As	649	4.06	2.68–6.81	< 0.30^b^	17.64	2.3
	Bi	649	0.02^b^	0.02^b^–0.02^b^	< 0.02^b^	2.51	92.6
	Cd	649	1.18	0.74–1.79	< 0.10^b^	11.23	5.4
	Cu	649	1289.2	1155.9–1449.1	501.5	2677.5	0
	Pb	649	10.83	8.65–13.50	3.10	70.24	0
	Sb	649	0.20	0.06^b^–0.40	< 0.06^b^	7.99	31.4
	Se	649	176.4	155.1–196.7	93.3	416.3	0
	Sn	649	0.20^b^	0.20^b^–0.38	< 0.20^b^	7.95	55.6
	THg^a^	650	5.42	3.89–7.59	0.61	25.19	0
	MeHg^a^	645	5.15	3.68–7.15	0.60	24.99	0
	IHg^a^	645	0.24	0.09–0.43	< 0.01	1.61	
	Zn	649	4769.0	4146.7–5417.8	2707.2	14,416.6	0
Cord blood (ng mL^−1^)	As	594	3.68	2.58–5.25	< 0.30^b^	22.41	0.5
	Bi	594	0.02^b^	0.02^b^–0.02^b^	< 0.02^b^	1.87	82.0
	Cd	594	0.53	0.10^b^–1.25	< 0.10^b^	10.52	26.4
	Cu	594	510.8	456.2–566.5	243.5	1429.8	0
	Pb	594	9.89	8.02–12.48	3.66	61.61	0
	Sb	594	0.39	0.06^b^–0.92	< 0.06^b^	6.40	28.3
	Se	594	191.4	166.5–219.0	73.9	376.2	0
	Sn	594	0.20^b^	0.20^b^–0.38	< 0.20^b^	5.23	63.6
	THg^a^	601	9.96	7.05–13.80	1.60	43.90	0
	MeHg^a^	598	9.47	6.70–13.28	1.52	43.15	0
	IHg^a^	598	0.27	0.10–0.63	< 0.01	2.43	
	Zn	594	2002.9	1757.7–2352.2	1103.9	22,258.6	0
Placenta (ng g-wet^-1^)	As	617	4.36	3.26–5.93	1.18	19.56	0
	Bi	617	0.03^b^	0.03^b^–0.04	< 0.03^b^	1.35	72.6
	Cd	617	16.95	12.97–22.72	3.52	51.49	0
	Cu	617	706.5	627.1–806.5	442.9	1419.3	0
	Pb	617	11.21	7.67–15.55	2.14	125.00	0
	Sb	617	0.24	0.10^b^–0.56	< 0.10^b^	33.43	37.8
	Se	617	295.3	265.0–331.9	172.4	503.5	0
	Sn	617	11.83	6.55–19.43	< 0.30^b^	197.56	1.9
	THg	617	12.60	9.31–16.47	1.98	52.44	0
	Zn	617	9101.4	8353.3–9917.1	6771.8	17,381.8	0

Copper, selenium and zinc concentrations were presented up to one decimal digit because of high concentration compared with other elemental concentrations

*As* arsenic, *Bi* bismuth, *Cd* cadmium, *Cu* copper, *Pb* lead, *Sb* antimony, *Se* selenium, *Sn* tin, *THg* total mercury, *MeHg* methylmercury, *IHg* inorganic mercury (IHg concentrations were calculated by subtracting MeHg from THg), *Zn* zinc

^a^ng g^−1^

^b^Limit of detection (LOD)

**Fig. 1:**
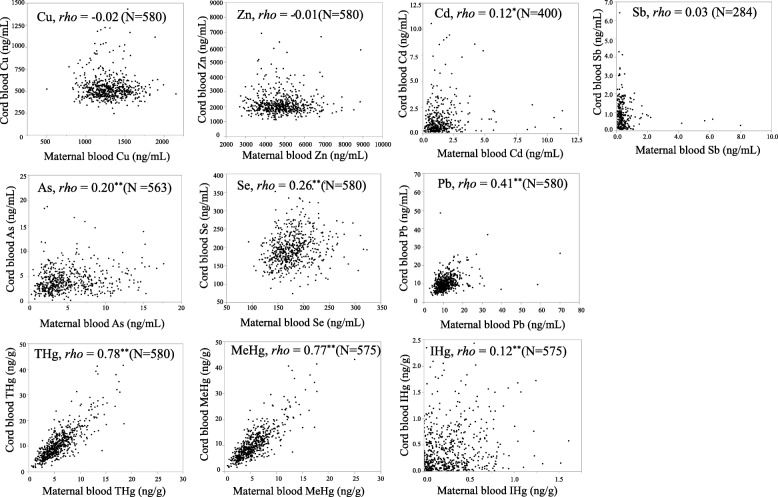
The relationships between the same element in the maternal blood and those in cord blood. Spearman’s rank correlation coefficients (*rho*), **P < 0.01, *P < 0.05. Values less than the LODs were excluded

Figure 1 displays simple correlations between each element in the maternal and cord blood. Values less than the LODs were also excluded. Strong correlations were observed between THg (and MeHg) in the maternal and cord blood (Spearman’s *rho* = 0.78 [0.77]). Correlations for Pb, As and Se in the maternal and cord blood were significant but moderate to weak (Spearman’s *rho* = 0.41, 0.20 and 0.26, respectively). No significant correlations were noted for Cu, Zn and Sb in the maternal and cord blood. Spearman’s correlations for the elements in the maternal blood, cord blood and placenta are shown as (Additional file 1: Table S3, *N* = 580).

**Fig. 2:**
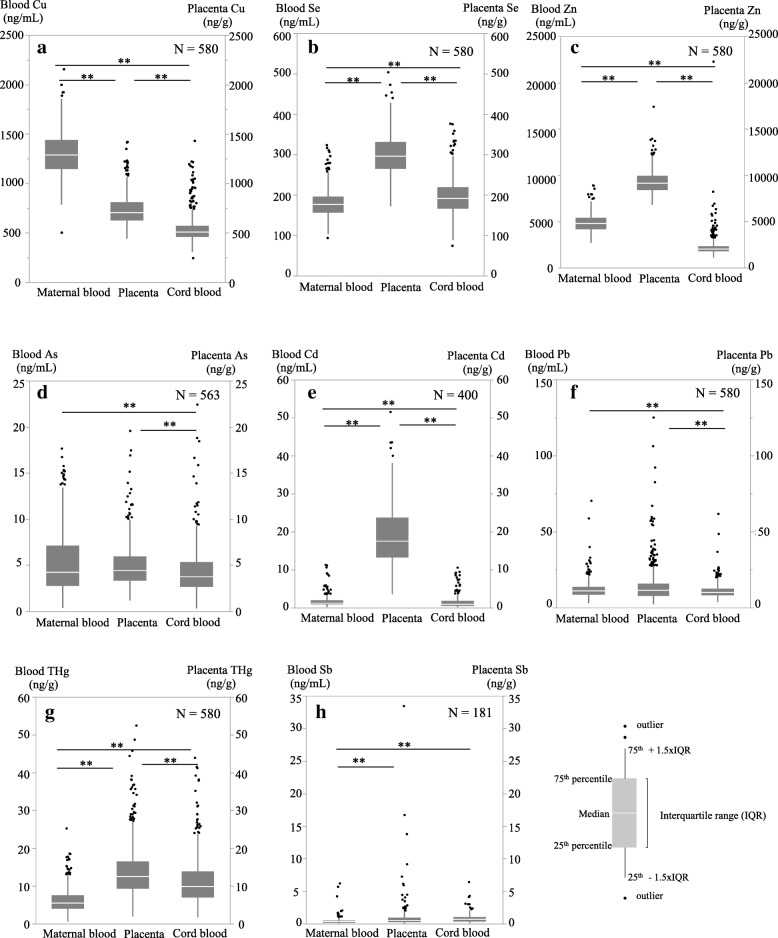
Box-plot showing the relationship of toxic metals among the maternal blood, cord blood and placenta. **a** Copper (Cu). **b** Selenium (Se). **c** Zinc (Zn). **d** Arsenic (As). **e** Cadmium (Cd). **f** Lead (Pb). **g** Total mercury (THg). **h** Antimony (Sb). The number of samples above LOD in the maternal blood, cord blood and placenta. Statistical analyses were performed by nonparametric Kruskal-Wallis followed by Dunn test. Significantly different (^**^P < 0.01), As and Pb: maternal blood (MB) and placenta (PL) > cord blood (CB), Cd: PL > MB > CB, Cu: MB > PL > CB, Se: PL > CB > MB, Sb: PL and CB > MB, THg: PL > CB > MB, Zn: PL > MB > CB

Figure 2 compares the concentrations of the toxic elements with the trace elements in the maternal blood, cord blood and placenta. Concentrations of As and Pb in the cord blood were significantly lower than those in the maternal blood and placenta. Concentrations of THg, Cd, Zn and Se in the placenta were significantly higher than those in the maternal and cord blood.

**Fig. 3:**
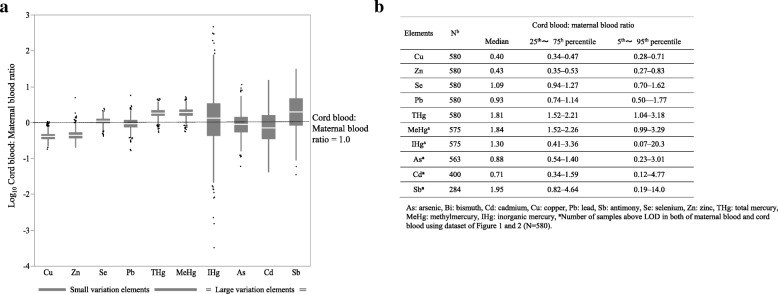
Box-plot and table showing cord blood to maternal blood ratios of toxic and trace elements in the study population. **a** Cord blood to maternal blood ratios of Cu, Zn, Se, Pb, THg and MeHg were small variation and those of IHg, As, Cd and Sb elements were large variation. **b** Table shows cord blood to maternal blood ratios of each element

Figure 3 shows cord blood to maternal blood ratios of the toxic and trace elements. The median ratios of Zn, Cu and Cd were approximately 0.5, and the median ratios of As, Se and Pb were approximately 1.0. By contrast, the median ratios of Sb, THg and MeHg were approximately 2.0. The variations of the Cu, Zn, Se, Pb, THg and MeHg ratios were small [relative standard deviation (RSD), 25–55%], whereas the variations of the As, Bi, Cd and Sb ratios were large (RSD, 94–450%).

## Discussion

MeHg and THg concentration levels in the cord blood showed strong associations with their concentration levels in the maternal blood, whereas As, Pb and Se concentration levels in the cord blood showed moderate associations with their concentration levels in the maternal blood. No significant associations were observed between the concentration levels of Cd, Zn, Cu and Sb in the maternal and cord blood. These findings indicate that Hg (i.e. MeHg and THg) concentrations in the maternal blood represent foetal exposure, whereas the others do not. Notably, even in the assessment of foetal exposure to MeHg, the cord blood is a more useful and valid biomarker [38]. This is so because the increased THg concentration in the cord blood was associated with some neurobehavioural and neurophysiological deficits in children [2, 38]. To assess the extent and resulting health effects of foetal exposure to As, Pb, Cd, Se, Zn, Cu and Sb, the use of the cord blood may be more suitable than the maternal blood (in especially, in case of no correlation between element concentrations in the maternal blood and cord blood). Maternal blood measurements have also the following some advantages: the maternal blood is easier to access, to get multiple sampling and more useful for preventive actions, including the process of setting reference values. To overcome this dilemma, foetal exposure simulation models must be developed using physiologically based pharmacokinetic (PBPK) modelling. The results of this study suggest that more information is required regarding the transplacental behaviour of these elements, such as active transportation and binding protein expressions [39, 40].

MeHg could be actively transported by the placenta through cysteine conjugate via the neutral amino acid carrier system [41]. Many studies have reported that MeHg was higher in the cord blood than in the maternal blood [42–45]. The average cord blood to maternal blood ratio of MeHg ranged from 0.8 to 2.8, with a mean value of 1.65 [45]. In this study, the median ratio was 1.8. Moreover, according to our review of the literature, this study may be the first to report the placental transfer of Sb (the median ratio was 2.0). The median cord blood to maternal blood ratios of Cd, Cu and Zn were less than 1 (there was no correlation between their element concentrations in the maternal and cord blood). Metallothionein of the metal-binding protein could be induced by some metals, such as Cd, Cu and Zn [46]. For a Cd and Zn interaction, the accumulation of Cd in the placenta reportedly induces the expression of metallothionein, which could lead to Zn retention in the placenta with subsequent reduced transfer to the foetus [47, 48]. This finding indicates that metallothionein expression in the placenta plays a role in modifying the transfer of Cd, Cu and Zn to the cord blood.

The median of blood Sb concentrations in a sample of 89 male and female German adults (median 0.6 μg L^−1^, range < LOD–7.54 μg L^−1^, [49]) was similar to the levels reported in this study, whereas these concentrations in pregnant women in Western Australia (median 1.54 μg L^−1^, range 0.16–7.31 μg L^−1^, [50]) were slightly higher in the participants of this study. Because maximum Sb levels in the participants in this study were similar to the levels noted in German adults and the pregnant women in Western Australia, we considered that the participants in this study were under general environmental Sb exposure. Reports have suggested that household dust [51], soil and airborne particles [52] are contaminated with Sb. Yoshinaga et al. [53] reported that geometric mean Sb levels (SD) in house dust of Japanese residences (*N* = 100) was 10.1 (2.06) mg kg-dry^−1^ and that result was two times higher levels than house dust in Canada. Further studies are necessary to evaluate the exposure source of Sb and the effects.

Kidney-Cd half-life was estimated between 18 and 44 years, whereas blood-Cd half-life was a few months [54, 55]. Certain studies have reported a higher accumulation of Cd in the placenta than in the maternal blood or cord blood because of the limited transplacental passage [56, 57]. This result could explain the comparatively no correlation between Cd levels in the maternal blood and cord blood (*r* = 0.042 [58], 0.25 [59], − 0.14 [42]). Compared with the placental Cd levels reported in 46 studies [60], the participants of this study are observed to have experienced moderate Cd exposure.

The geometric mean levels of blood THg were 0.678 ng mL^−1^ in NHANES 2013–2014 (*N* = 2628, female) in the United States (USA) [61]. The median levels of maternal blood THg were 0.64 ng mL^−1^ in MIREC (*N* = 1835) in Canada [62] and 2.24 ng mL^−1^ in Taiwan (*N* = 145, [63]). Our median value of 5.42 ng g^−1^ (5.69 ng mL^−1^, adjusted for blood-specific gravity as 1.05) in the maternal blood was higher than the median values obtained from these countries. The mercury exposure of the participants in this study was lower compared with the participants in the Faroese birth cohort study and the Seychelles Child Development Study [2].

The geometric mean levels of Pb in the blood were 0.842 μg dL^−1^ in NHANES 2013–2014 (*N* = 2628, female) in the USA [61] and 3.97 μg dL^−1^ in China (*N* = 1931, [64]). The median levels of maternal blood Pb were 0.59 μg dL^−1^ in MIREC (*N* = 1835) in Canada [62]. Our median value of 1.08 μg dL^−1^ in the maternal blood is similar to these values, with the exception of China.

The cord blood As level in this study was similar to that obtained from the USA (child blood, [65]) and Taiwan (cord blood) and was a little higher than that from Nepal (cord blood) [66, 67]. Because we measured only total As levels in the blood, we do not have data regarding inorganic As (and the metabolites) and high toxicity (our limitation). A significant number of studies have measured total As and/or inorganic As by speciation using urine [66]. Individuals in Japan are exposed to organic As, especially arsenobetaine, the nontoxic form of As, which is typically found in seafood, especially shellfish and seaweed [68, 69]. Notably, the positive relationship between THg and As (details are presented in Additional file 1: Table S3) may be a result of seafood consumption. Exposure to As is typically measured by analysing urine and blood samples [70]. To investigate the effects of inorganic As on child development, we must assess the extent of exposure to inorganic As by speciation. By doing so, we can determine the inorganic As level in the cord blood; hair and nails are also reliable indicators of long-term exposure to inorganic As [71].

### Elements with a small variation in cord blood to maternal blood ratios (Cu, Zn, Se, Pb, THg and MeHg)

The variations in cord blood to maternal blood ratios among individuals were low for Cu, Zn and Se (RSD = 32, 55 and 25%, respectively). Cu, Zn and Se are essential trace elements involved in many life-supporting biochemical processes [17]. By contrast, excess intake of these trace elements leads to disease and toxicity [17]. Notably, because Cu and Zn are highly toxic for the foetus [72], cord blood Cu (about 500 ng mL^−1^ in median) and cord blood Zn (about 2000 ng mL^−1^ in median) seemed to be properly regulated in vivo (details in Fig. 2).

The variations in ratios among individuals were also low for the following toxic metals: Pb, THg and MeHg (RSD = 43, 33 and 34%, respectively). It may be regulated in vivo like essential trace elements. Although Pb is not essential to support life, it is freely transported from the mother to the foetus through the placenta [60]. A possibility is that Pb^2+^ ions mimic Ca^2+^ ions [73] in several physiological phenomena. For example, maternal dietary calcium supplementation during pregnancy and lactation was associated with reductions in Pb levels in animal and human studies [74].

### Elements with large variations in cord blood to maternal blood ratios (As, Cd and Sb)

The variations in cord blood to maternal ratios among individuals were high for As, Sb and Cd (RSD = 94, 130 and 130%, respectively), as exists in different chemical forms in the blood. The proportion of the different As chemical species may largely vary among individuals, which may have resulted in the large variation in As ratios. A speciation analysis of As is essential to assess the characterisation of its transplacental behaviour and developmental effects [13]. Sb cord blood to maternal blood ratios exceeding 1 were observed in 71% of the participants. Because the Sb concentrations were at low levels in the cord blood and maternal blood, the large variation of the Sb ratio might include the uncertainty of the analytical values (limitation). However, the Sb cord blood to maternal blood ratio was 2.0, and this level of exposure to Sb would be problematic for foetuses from a health perspective.

Moreover, cord blood to maternal blood Cd ratios > 1 were observed in 39% of the participants, with large variation among individuals. Genetic variation leading to differences in expression and regulation of metallothionein proteins may have contributed to the differences observed among participants in terms of Cd uptake and metabolism [75]. Further research should focus on genetic variation analyses such as single nucleotide polymorphisms (SNPs) to identify vulnerable sections of the population.

There are some limitations of our research. Firstly, there was time-lag between sampling of the maternal blood and cord blood in the urban area of our cohort. Copper, zinc and selenium are essential trace elements of which blood concentrations are maintained in certain ranges. Estimated daily dietary intakes of total zinc were 8.8–14.4 mg/day for adults aged 20–50 years [76]. The average daily intake of copper was about 1 mg with the primary source being the diet [77]. We could assume that blood trace element concentrations were in steady states since they were taken daily via diet. Actually, Willett reported that observed intra-class correlations (ICC) in 3-month intervals were 0.76 for serum selenium and 0.95 for whole blood selenium. This suggests that whole blood selenium concentration is less susceptible to temporal variation and thus a better index of long-term intake compared to the serum selenium concentration [78]. Lee at al. reported that whole blood ICC for Pb, Hg and Cd was 0.81, 0.71 and 0.83, respectively [79]. We could not find about blood ICC of other elements. We might be able to confirm the effect of sampling time-lag on the cord/maternal blood ratio using the sample of coastal area in our cohort because sampling of the maternal blood (1 day postpartum) in the coastal area was almost same with the cord blood (at birth). Secondly, in case some element concentrations were extremely of low levels in the cord blood and maternal blood, the large variation of the ratio might include the uncertainty of the analytical values. Thirdly, we had not performed arsenic speciation yet. We would conduct further research about arsenic speciation in the future using a sample of our cohort. Lastly, this study has certain limitations because we do not have data regarding genetic background, such as SNPs and DNA methylation. A noticeable limitation is we could not reveal the effects of a genetic factor on the large variations in the ratio between individuals.

## Conclusions

Sb was detected in 72% of the cord blood samples, whereas Sn was detected in 44% of the maternal blood samples. By contrast, Bi was detected in 7% of the maternal blood samples. Sn was detected in 36% of the cord blood samples, whereas Bi was detected in 18% of the cord blood samples. Concentrations of Zn, Cu, Pb, Cd and As in the maternal blood were significantly higher than their concentration levels in the cord blood. In contrast, THg, MeHg and Sb levels in the cord blood were approximately two times higher than their levels in the maternal blood. Foetal exposure to elements with large variations in the cord blood to maternal blood ratios (IHg, As, Cd and Sb) should be evaluated using the cord blood samples, especially because the placental transfer of these elements varies largely among individuals. In the future, our results will be useful to evaluate the exposure levels of these elements and to investigate the associations between toxic element exposure and children’s health [80].

## Additional file

Additional file 1: **Table S1.** Results from analytical quality control of toxic and trace elements in the whole blood. **Table S2.** Results from external quality control and other analytical reference values of toxic and trace elements in the whole blood. **Table S3.** Spearman’s rank correlation coefficients (*rho*) for five elements in the maternal blood, cord blood and placenta. **Figure S1.** Flow chart of the study participants. **Figure S2.** Correlations of blood mercury concentrations between A laboratory (International mercury lab) and B laboratory (IDEA consultant). *N* = 5, Pearson product-moment correlation coefficient (*r*). A (total mercury in the whole blood): *Y* = 0.96*x* − 0.99, B (methylmercury in the whole blood): *Y* = 0.97*x* − 1.1, C (total mercury in red blood cells): *Y* = 0.84*x* + 0.48, D (methylmercury in red blood cells): *Y* = 0.82*x* − 0.83, E (total mercury in plasma): *Y* = 0.77*x* + 0.21, F (methylmercury in plasma): *Y* = 0.65*x* + 0.08. (PDF 275 kb)

## Abbreviations

ADHD: Attention-deficit/hyperactivity disorder
As: Arsenic
Bi: Bismuth
Cd: Cadmium
Cu: Copper
CVAAS: Cold vapour atomic absorption spectrometry
GC-ECD: Gas chromatography with electron capture detection
Hg: Mercury
ICC: Intra-class correlations
ICP-MS: Inductively coupled plasma mass spectrometry
IHg: Inorganic mercury
LOD: Limit of detection
MeHg: Methylmercury
Pb: Lead
RSD: Relative standard deviation
Sb: Antimony
Se: Selenium
Sn: Tin
SNPs: Single nucleotide polymorphisms
THg: Total mercury
TSCD: Tohoku Study of Child Development
Zn: Zinc
