# Supplementary material for: Exposure profile of mercury, lead, cadmium, arsenic, antimony, copper, selenium and zinc in maternal blood, cord blood and placenta: the Tohoku Study of Child Development in Japan
Source: Environ Health Prev Med. 2019 May 17;24:35. doi: 10.1186/s12199-019-0783-y (PMC6525413; doi:10.1186/s12199-019-0783-y)
Supplement: Supplementary file 1 — Table S1. Results from analytical quality control of toxic and trace elements in the whole blood. Table S2. Results from external quality control and other analytical reference values of toxic and trace elements in the whole blood. Table S3. Spearman’s rank correlation coefficients (rho) for five elements in the maternal blood, cord blood and placenta. Figure S1. Flow chart of the study participants. Figure S2. Correlations of blood mercury concentrations between A laboratory (International mercury lab) and B laboratory (IDEA consultant). N = 5, Pearson product-moment correlation coefficient (r). A (total mercury in the whole blood): Y = 0.96x − 0.99, B (methylmercury in the whole blood): Y = 0.97x − 1.1, C (total mercury in red blood cells): Y = 0.84x + 0.48, D (methylmercury in red blood cells): Y = 0.82x − 0.83, E (total mercury in plasma): Y = 0.77x + 0.21, F (methylmercury in plasma): Y = 0.65x + 0.08. (PDF 275 kb) [file 12199_2019_783_MOESM1_ESM.pdf]

Supplementary data for

Exposure profile of mercury, lead, cadmium, arsenic, antimony, copper, selenium and zinc in maternal blood, cord blood and placenta: the Tohoku Study of Child Development in Japan

Miyuki Iwai-Shimada[1], Satomi Kameo[2], Kunihiko Nakai[3], Kozue Yaginuma-Sakurai[2], Nozomi Tatsuta[3], Naoyuki Kurokawa[2], Shoji F. Nakayama[1], Hiroshi Satoh[4]

[1]National Institute for Environmental Studies, Tsukuba, Ibaraki, Japan

[2]Environmental Health Sciences, Tohoku University Graduate School of Medicine, Sendai, Miyagi, Japan

[3]Department of Development and Environmental Medicine, Tohoku University Graduate School of Medicine, Sendai, Miyagi, Japan

[4]Professor Emeritus, Tohoku University, Sendai, Miyagi, Japan

*Corresponding author: Miyuki Iwai-Shimada

This file includes:

Table S1. Results from analytical quality control of toxic and trace elements in whole blood.

Table S2. Results from external quality control and other analytical reference value of toxic and trace elements in whole blood

Table S3. Spearman's rank correlation coefficients (rho) for five elements in maternal blood, cord blood and placenta.

Figure S1. Flow chart of Study participants

Figure S2. Correlations of blood mercury concentrations between A laboratory (International mercury lab) and B laboratory (IDEA consultant). N=5, Pearson product-moment correlation coefficient (r). A (total mercury in whole blood): Y=0.96x-0.99, B (methylmercury in whole blood): Y=0.97x-1.1, C (total mercury in red blood cells): Y=0.84x+0.48, D (methylmercury in red blood cells): Y=0.82x-0.83, E (total mercury in plasma): Y=0.77x+0.21, F(methylmercury in plasma): Y=0.65x+0.08.

Supplementary data

Table S1. Results from analytical quality control of toxic and trace elements in whole blood

Elements	Average analytical value (S.D.)	RSD[a] (%)	Our methods	N[b]	Seronorm whole blood level (Lot)	Acceptable range (reference value)
**As**	13.50 (0.63) ng mL^{-1}	4.6	ICP-MS	30	Level 2 (0503109)	10.6–15.8 (13.2)
**Bi**	5.04 (0.14) ng mL^{-1}	2.8	ICP-MS	29		4.5–5.7 (5.1)
**Cd**	6.07 (0.28) ng mL^{-1}	4.6	ICP-MS	29		5.2–6.8 (6.0)
**Se**	120.1 (7.77) ng mL^{-1}	6.5	ICP-MS	29		103–143 (123)
**Sb**	30.95 (0.81) ng mL^{-1}	2.6	ICP-MS	29		25.3–32.9 (29.1)
**Pb**	406.3 (8.03) ng mL^{-1}	2.0	ICP-MS	29		351–435 (393)
**Cu**	626.8 (27.1) ng mL^{-1}	4.3	ICP-MS	29		623[c]
**Zn**	5356.7 (255) ng mL^{-1}	4.8	ICP-MS	29		5216[c]
**THg**	12.8 (0.7) ng g^{-1}	5.5	CVAAS	43	Level 3 (OK0337)	12.3–13.7 (13.0)

As: arsenic, Bi: bismuth, Cd: cadmium, Cu: copper, Pb: lead, Sb: antimony, Se: selenium, THg: total mercury and Zn: zinc, [a]relative standard deviation, [b]Number of experiments; reference materials were analysed one time per day. [c]Approximate value.

Table S2. Results from external quality control and other analytical reference value of toxic and trace elements in whole blood

	Elements	Analytical value	Acceptable range (reference value)
**Seronorm whole blood Level 1**	Cd	0.76 ng mL^{-1}	0.68-0.80 (0.74)
	Sn	0.35 ng mL^{-1}	0.29-0.39 (0.34)
	Sb	1.6 ng mL^{-1}	1.5-1.7(1.6)
	Pb	27.3 ng mL^{-1}	26.2-29.0 (27.6)
	Cu	540 ng mL^{-1}	531-597 (564)
	Zn	5357 ng mL^{-1}	5200-5800 (5500)
**G-EQUAS**	Pb	A: 624.2 ng mL^{-1}<br>B: 266.5<br>C: 99.8<br>D: 51.3	A: 563.6-717.4 (640.5) [a]<br>B: 239.2-327.6 (283.4) [a]<br>C: 93.59-126.62 (110.11) [a]<br>D: 45.9-64.84 (55.37) [a]
	Cd	A: 14.1 ng mL^{-1}<br>B: 4.6<br>C: 1.10<br>D: 0.40	A: 11.6-16.1 (13.9) [a]<br>B: 3.5-5.4 (4.5) [a]<br>C: 0.77-1.44 (1.11) [a]<br>D:0.23-0.51 (0.37) [a]

G-EQUAS: German External Quality Assessment Scheme, Cd: cadmium, Cu: copper, Sn: tin, Sb: antimony, Pb: lead and Zn: zinc, [a]tolerance range.

Table S3. Spearman's rank correlation coefficients (*rho*) for five elements in maternal blood, cord blood and placenta.

N = 580		Maternal blood							Cord blood							Placenta						
		As	Cd	Cu	Pb	Se	THg	Zn	As	Cd	Cu	Pb	Se	THg	Zn	As	Cd	Cu	Pb	Se	THg	Zn
Maternal blood	As	1.00	0.05	0.16^{*}	0.04	0.21^{**}	0.24^{**}	0.16^{**}	0.21^{**}	0.02	-0.08	-0.04	-0.10^{*}	0.21^{**}	-0.07	0.27^{**}	0.11^{*}	0.04	0.06	0.10^{*}	0.22^{**}	0.03
	Cd		1.00	-0.09^{*}	0.25^{**}	0.16^{**}	0.11^{**}	0.16^{**}	-0.03	0.08	0.15^{**}	0.07	-0.08	-0.01	0.07	-0.01	0.27^{**}	0.11^{**}	-0.02	0.08	0.04	0.08
	Cu			1.00	-0.06	0.14^{**}	-0.01	0.15^{**}	0.15^{**}	0.02	-0.02	-0.11^{**}	-0.03	0.02	-0.07	0.08	-0.01	0.14^{**}	-0.04	-0.10^{*}	0.01	-0.11^{**}
	Pb				1.00	0.14^{**}	0.17^{**}	0.23^{**}	0.08^{*}	0.00	0.13^{**}	0.41^{**}	0.03	0.02	0.07	0.03	0.21^{**}	0.07	0.14^{**}	0.01	0.07	0.06
	Se					1.00	0.28^{**}	0.48^{**}	0.04	-0.01	0.01	0.03	0.26^{**}	0.16^{**}	-0.04	0.03	-0.02	0.06	0.04	0.20^{**}	0.17^{**}	0.05
	THg						1.00	0.20^{**}	0.21^{**}	0.04	0.00	0.08	0.04	0.78^{**}	0.04	0.17^{**}	0.09^{*}	0.06	-0.02	0.01	0.80^{**}	0.02
	Zn							1.00	0.06	0.00	-0.07	-0.07	-0.02	0.05	-0.01	0.01	-0.02	-0.01	0.04	-0.07	0.05	-0.02
Cord blood	As								1.00	-0.05	0.06	0.05	0.13^{**}	0.26^{**}	0.20^{**}	0.57^{**}	0.08	0.02	0.05	-0.11^{**}	0.25^{**}	-0.04
	Cd									1.00	0.11^{**}	0.15^{**}	-0.06	-0.04	0.15^{**}	0.04	0.07	0.02	-0.09^{*}	0.10^{*}	0.05	0.06
	Cu										1.00	0.26^{**}	0.12^{**}	-0.05	0.41^{**}	-0.04	0.02	0.12^{**}	0.01	0.04	0.00	-0.03
	Pb											1.00	0.09^{*}	0.09^{*}	0.25^{**}	0.03	0.22^{**}	0.11^{**}	0.23^{**}	0.03	0.07	0.11^{**}
	Se												1.00	0.21^{**}	0.00	0.00	-0.09^{*}	0.02	0.03	0.27^{**}	0.06	0.04
	THg													1.00	-0.03	0.18^{**}	0.09^{*}	0.06	0.02	0.02	0.85^{**}	-0.02
	Zn			-											1.00	0.04	0.01	0.04	0.02	0.00	0.03	0.02
Placenta	As															1.00	0.21^{**}	0.13^{**}	0.03	0.08	0.23^{**}	0.12^{**}
	Cd																1.00	0.18^{**}	0.13^{*}	0.02	0.08	0.15^{**}
	Cu																	1.00	0.09^{*}	0.28^{**}	0.11^{*}	0.23^{**}
	Pb																		1.00	-0.05	-0.03	0.20^{**}
	Se																			1.00	0.09^{*}	0.43^{**}
	THg																				1.00	0.07
	Zn																					1.00

As: arsenic, Cd: cadmium, Cu: copper, Pb: lead, Se: selenium, THg: total mercury and Zn: zinc, Values are Spearman's rank correlation coefficients (*rho*), $P < 0.01$**, $P < 0.05$*. For the undetected samples of As and Cd, values of one half (1/2) of the limit of detection (LOD) were assigned (N = 580).

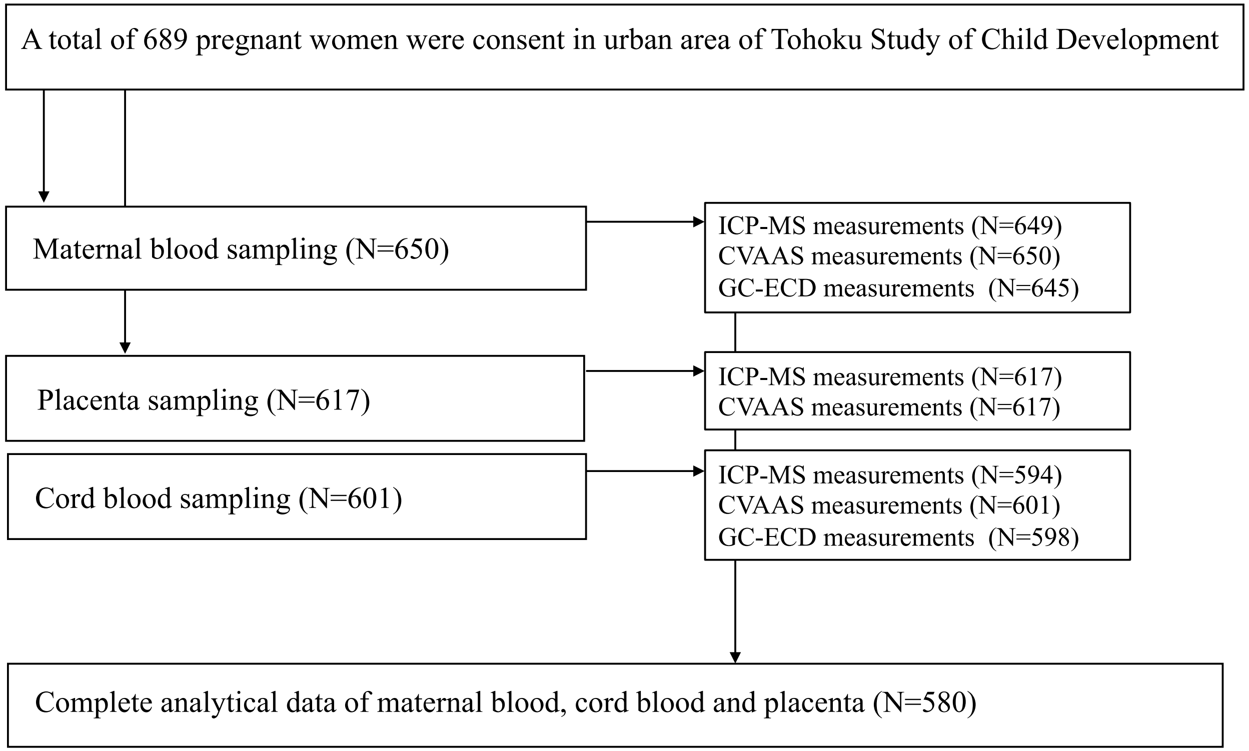

Figure S1. Flow chart of Study participants

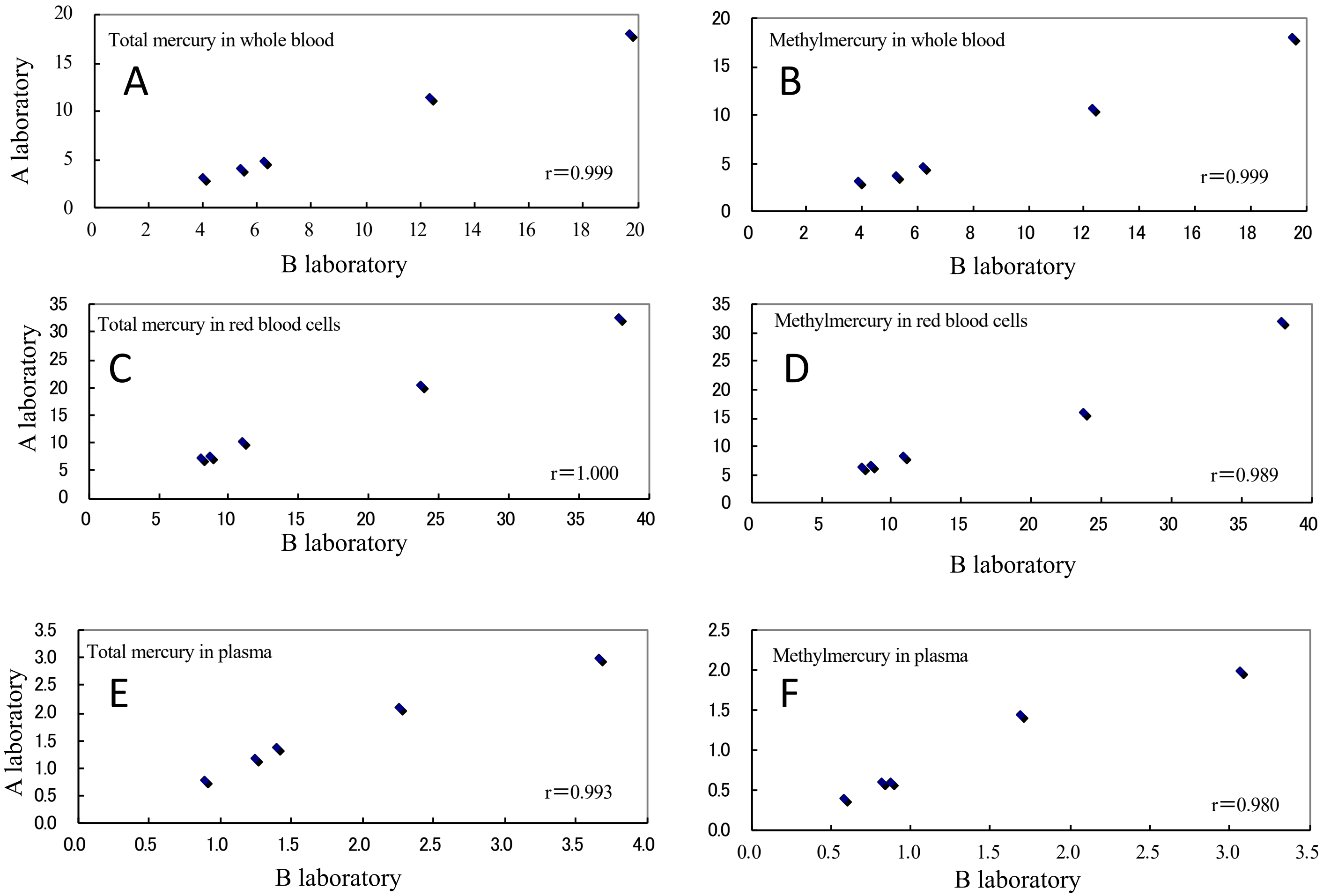

Figure S2. Correlations of blood mercury concentrations between A laboratory (International mercury lab) and B laboratory (IDEA consultant). N=5, Pearson product-moment correlation coefficient (r). A (total mercury in whole blood): Y=0.96x-0.99, B (methylmercury in whole blood): Y=0.97x-1.1, C (total mercury in red blood cells): Y=0.84x+0.48, D (methylmercury in red blood cells): Y=0.82x-0.83, E (total mercury in plasma): Y=0.77x+0.21, F(methylmercury in plasma): Y=0.65x+0.08.
